# “Bariatric families”- a new phenomenon with unique characteristics

**DOI:** 10.1186/s12887-020-02226-2

**Published:** 2020-07-02

**Authors:** Netta Weiss, Nataly Kalamitzky, Hagar Interator, Ronit Lubetzky, Hadar Moran-Lev

**Affiliations:** 1 Department of Pediatrics, Dana Dwek Children’s Hospital, Tel Aviv, Israel; 2 Department of Pediatric Gastroenterology, Dana Dwek Children’s Hospital, Tel Aviv, Israel; 3 affiliated to the Sackler Faculty of Medicine, Tel Aviv University, Tel Aviv, Israel

**Keywords:** Bariatric surgery, Obesity, Health perception

## Abstract

**Background:**

Many obese children have at least one obese parent, and some of them have one parent who had undergone bariatric surgery (“bariatric families”). The perceptions and attitudes towards child obesity of parents in bariatric families vs. non-bariatric families have not been explored. We assessed how parents who underwent bariatric surgery for obesity perceived their child’s obesity compared to those perceptions of obese parents who did not undergo bariatric surgery.

**Methods:**

We conducted a cross-sectional survey by interviewing families in which one or both parents underwent bariatric surgery (bariatric group) and comparing their responses to those of families in which one or both parents had been treated conservatively for obesity (control group). The children of both groups were attending the Obesity Clinic of our children’s hospital.

**Results:**

Thirty-six children (median age 10.6 years, 18 in each group, matched for age and sex) were recruited. More parents in the bariatric group replied that weight plays an important role in determining self-image (*p* < 0.03), and more replied that their child’s obesity is a current and future health problem (*p* < 0.03 and *p* < 0.007, respectively, Table 1). Five children (28%) in the bariatric group had expectations of undergoing bariatric surgery compared to none in the control group (*p* < 0.02), with a similar trend among their parents (44% vs. 11%, respectively, *p* < 0.07).

**Conclusion:**

Families in which one or both parents underwent bariatric surgery for obesity revealed different perceptions of their child’s obesity and different opinions about interventions for treating it compared to families with no bariatric surgery.

## Background

Obesity has become a major public health problem of global significance [1]. The prevalence of obesity in the general population has skyrocketed during the past 20 years, having more than doubled in children and quadrupled in adolescents in the past 30 years. Between 2011 and 2014, an estimated 17% of all US children between 2 and 19 years were obese [2]. Childhood obesity increases the risk of obesity in older age and is strongly associated with significant short- and long-term adverse medical and psychosocial effects [3, 4]. Despite the rising numbers, the results of treatment for obesity have been generally unsatisfactory. Several studies have observed high dropout rates and only modest weight loss among those who maintained long follow-up [1, 5, 6].

Weight loss by means of surgical treatment for severely obese adolescents is still considered to be controversial by healthcare professionals. Nonetheless, surgical weight loss presents an alternative that could be offered to morbidly obese youngsters with serious comorbidities, such as obstructive sleep apnea or non-alcoholic fatty liver with fibrosis [7].

Family attitudes were reported as being a significant mediator in children’s weight loss treatment effectiveness [8, 9]. The parents’ concept of pediatric obesity had significant influence on their opinion of bariatric surgery. Specifically, parents who felt that obesity was inevitable for them or their children were more inclined to consider bariatric surgery an appropriate solution to their child’s obesity [10]. In contrast, parents who thought they could influence their child’s obesity were more in favor of non-surgical treatment [11].

Obesity is often familial, and a large number of obese children have at least one obese parent. In some of those families, one parent or both had undergone bariatric surgery ("bariatric families). Bariatric parents’ perceptions of their child’s obesity and of the treatment options available to the child have not been documented, nor have those perceptions of obese non-bariatric parents. The aim of the present study is to assess these perceptions as well as those of the obese children among both groups of parents.

## Methods

### Patient population

The study was performed in the Nutrition and Obesity Clinic at “Dana Dwek” Children’s Hospital. Of the 489 families who visited the clinic between January 1, 2011 and January 1, 2017, 352 had an obese child aged 1–18 years. Eighteen of them were “bariatric families” in which at least one parent had undergone bariatric surgery (study group). A control group matched by age and gender was selected from the remaining 334 families. Patients with missing or incomplete data were excluded from entering the study. None of the participants refused to enroll in the study.

### Study design

Sociodemographic, clinical, and anthropometric data were collected from the medical records of all participants. They were also contacted by telephone by one investigator (N.W) and completed a questionnaire designed for gathering data for this study. It contained 27 questions and was based on two validated sources: The Levenson Multidimensional Locus of Control Scales, which assess how a person perceives his life (internal or external control) [12], and The Pediatric Quality of Life Inventory (PedsQL), that measures health-related quality of life in children and adolescents [13]. These items were supplemented by an original questionnaire aimed at assessing specific perceptions of obesity in bariatric families. Our questionnaire examined three dimensions of the parental perception of their child’s weight: (1) overweight as a deterministic factor, (2) health and psychosocial consequences of overweight, and (3) the ability to make changes and to lose weight. The participants were asked to either rate their answer on a scale of 1 (I absolutely disagree) to 5 (I absolutely agree), or to choose between yes/no/not relevant. Academic education of the parents was defined as “yes” if the parent had 12 or more years of education. Normal parental BMI was defined as a BMI between 18 and 25. A parent was defined as maintaining a healthy lifestyle if it included regular exercise and the consumption of healthy food.

The obese children’s perceptions of their health and social status were documented during their first encounter at the clinic on a scale of 1 (worst) to 10 (best). Those data were retrieved from their medical records.

### Analysis

The statistical analysis was performed with SPSS (IBMSPSS statistics, version 22, IBM Corp. Armonk, NY, USA, 2013.). Descriptive statistics were examined for all variables. Continuous variables were expressed as median with interquartile range (IQR) when they were not normally distributed and as mean ± standard deviation (SD) for normally distributed variables. Categorical variables were presented as number and percentage. All statistical tests were two-sided. Categorical variables were compared by the McNamer test and continuous and ordinal variables by the Wilcoxon test. When McNamer test was not applicable for some variables the Fischer test was used instead. A *p* level < 0.05 was considered statistically significant. Internal consistency of the questionnaire was evaluated using alfa Cronbach.

### Ethical considerations

The study protocol was approved by the institutional review board of the Tel Aviv Sourasky Medical Center (TLV-0097-17). Signed informed consent was obtained from the parents of all the participants.

## Results

The demographic and lifestyle characteristics of all the children and parents who participated in this study are listed in Table 1. Eighteen children were recruited to the bariatric group and 25 children who were matched for age and sex were recruited to the control group, of whom seven were excluded due to incomplete data, leaving 18 children in the control group. The median age of the children in both groups was 10.6 years, and 61% were females. The body mass index (BMI) values of all the children were in the obese range (median BMI 32.3 for the bariatric group and 28.6 for the control group). There were no group differences in mean birth weight, high-risk pregnancies, developmental delays, or previous hospitalizations, as well as in parental characteristics. However, the children in the bariatric group reported significantly more trials to lose weight in the past (72% vs. 44% for the controls, *p* < 0.02).

**Table 1:** Demographic and Clinical Characteristics of the Study Children and Parents

Characteristics	Bariatric group	Control group	***p*** value
**Children**			
Mean age, years (range)	10.6 ± 3.7 (2–16)	10.7 ± 3.5 (3.5–16)	NS
Sex (M:F)	7:11	7:11	NS
BMI (range)	32.32 ± 8.55 (22.15–49)	28.59 ± 5.5 (20.1–40.9)	NS
Mean birth weight, gr (range)	3281 ± 609 (1800–4500)	3091 ± 760 (825–4200)	NS
High-risk pregnancy n, (%)	2 (11%)	0 (0%)	NS
Developmental delay n, (%)	7 (38%)	6 (33%)	NS
Previous hospitalization n, (%)	4 (22%)	5 (28%)	NS
Previous trials to lose weight n, (%)	13 (72%)	8 (44%)	0.02
Academic education – father yes n (%)	7 (41%)	11 (61%)	NS
Academic education – mother yes n (%)	9 (50%)	13 (76%)	NS
Divorced parents n, (%)	3 (16.7%)	4 (22.2%)	NS
Healthy lifestyle - father n, (%)	6 (33.3%)	9 (50%)	NS
Healthy lifestyle - mother n, (%)	3 (17%)	8 (47.1%)	NS
Healthy BMI - father n, (%)	8 (44%)	10 (88%)	NS
Healthy BMI - mother n, (%)	7 (39%)	10 (59%)	NS
Obese family member n, (%)	18 (100%)	12 (67%)	0.02

*BMI* body mass index

The responses that reflected the obese child’s perspective are given in Table 2. There were no group differences in the child’s ranking of academic and social skills. The children’s reported motivation to lose weight was similar in both groups, as was their estimated body image. There was, however, a significant difference in the children’s willingness to undergo a bariatric procedure: 5 (28%) of the children in the bariatric group were interested in bariatric surgery instead of a conservative approach to losing weight compared to none of the children in the control group (*p* < 0.02).

**Table 2:** Children’s Psychosocial View of Themselves and Interest in Bariatric Surgery

Variable	Bariatric group	Control group	***p*** value
Child’s self-estimated body image (1–10)	4.63 ± 2.6 (1–10)	4.93 ± 2.3 (1–8)	NS
Child’s self-estimated social functioning (1–10)	8.71 ± 1.8 (5–10)	7.83 ± 2.4 (3–10)	NS
Child’s self-estimated academic skills (1–10)	8.08 ± 2.11 (4–10)	6.79 ± 1.37 (5–9)	NS
Child’s self-estimated motivation to lose weight (scale 1–10)	8.73 ± 1.73 (5–10)	8.08 ± 1.89 (5–10)	NS
Child’s interest in undergoing bariatric surgery	5 (28%)	None	0.02

Some differences were found in the parents’ psychosocial perception of their children’s weight and health-related consequences (Table 3). The parents in the bariatric group were more likely to think that the number of friends their child had was related to their child’s weight (*p* < 0.03). In addition, those parents were more likely to consider that their child’s weight played an important role in the child’s self- image (*p* < 0.03). The parents in the bariatric group were also more likely to think that their child will not attain a normal weight without an intervention (*p* < 0.02). The parents in the bariatric group were more likely to see their child’s weight as a health problem (*p* < 0.04). They were also more likely to fear that obesity is putting their child’s health at risk, but this difference did not reach statistical significance. The parents in both groups feared that their children will be overweight in the future, however, none of the parents’ responses indicated that they were worried about an obesity-related comorbidity.

**Table 3:** Parental Psychosocial and Health Consequence Perceptions

Item (answer in scale of 1–5)	Bariatric group^a^	Control group^a^	***p*** value
My child’s weight influences his/her self-image	4.28	3.67	0.03
The number of friends my child has is related to his/her weight	2.56	1.78	0.03
My child needs outside intervention in order to lose weight	4.39	3.56	0.02
Weight is determined by fate	2.00	1.82	NS
Exercising regularly is needed in order to lose weight	4.5	4.44	NS
Healthy nutrition is needed in order to lose weight	4.72	4.67	NS
My child’s weight puts his/her current health at risk	4.06	2.89	0.04
My child’s weight puts his/her future health at risk	4.89	3.78	0.07
I’m worried that my child will by overweight in the future.	4.5	4.11	NS
Does your child’s weight put him/her at risk of the comorbidities of obesity? YES/NO (%)	2 (11%)/16 (89%)	3 (17%)/15 (83%)	NS

^a^Mean values

Most of the participants agreed that overweight is not a deterministic factor: 72% of the parents in the bariatric group and 77% of the parents in the control group disagreed that weight is determined by fate. There was unanimous agreement about the parameter of lifestyle: they all responded that healthy nutrition and frequent physical activity are needed in order to lose weight.

The parental responses to the items on future intervention revealed that the parents in the bariatric group were four times more likely to think that their children will need a bariatric procedure in the future (44% vs. 11% for the controls, *p* < 0.07). Importantly, most of the parents who underwent bariatric surgery (83%) were satisfied with the surgery, considered it successful (72%), and would have done it again (83%).

## Discussion

This study compared the perceptions of the child’s obesity among families in which one or both parents underwent bariatric surgery to those of matched families without parental bariatric surgeries who served as controls. The perceptions of the obese children of both parental groups that were documented during their first encounter at the clinic were also compared. The bariatric parents were more inclined to think that their child’s obesity negatively affects his/her self-image and social status. They also tended to think that their child’s obesity is a current and future health problem, and that he/she will eventually need to undergo a bariatric procedure. The children in the bariatric group were more willing to undergo bariatric surgery themselves compared to the children in the control group.

Parental overweight is frequently cited as a predictor for childhood obesity [14, 15]. The familial effect on childhood obesity is multifactorial and has both genetic and environmental components [16]. The genetic component of obesity includes both differences in metabolic rate but more importantly the genetic of eating behavior which have an influence on meal timing, quantity of food intake, and food preference [15, 17]. Moreover, there is also an undeniable environmental component that comprised of food availability within the home, family meal structure, cultural preference, and poor engagement in exercise, maintains adolescent obesity [9, 18]. Several studies have illustrated the interdependence of home food availability and the intake of both unhealthy and healthy foods [19, 20], while other studies focused on the role of an obesogenic environment that promotes physical inactivity and access to energy-dense foods [21, 22]. As all the bariatric group parents faced morbid obesity, their households may have genetic and environmental influences their children’s weight and eating behaviors. Beyond the influence of the family on the tendency to become obese, it also has an effect on the attitude towards obesity. The bariatric parents are highly aware of the difficulties involved in the conventional methods of losing weight, especially considering their own failures to do so. This may cause them to be sceptical about their children’s chances to succeed. Van Geelen et al. assessed attitudes and normative beliefs about pediatric bariatric surgery among morbidly obese adolescents and parents [11]. Those authors showed that parents and adolescents who viewed obesity as something that they could influence themselves were more in favor of non-surgical treatment and vice versa. Given the bariatric parents’ life experiences and the fact they consider their own surgery as having been successful, it is understandable that they might want the same option for their children.

Thompson et al. have shown that different variables can influence the preferences of methods to lose weight, such as the number of attempts to lose weight, gender, age, BMI, and medical condition [23]. The present study extends these results and demonstrates that a history of bariatric surgery in the family can also have an impact of the perception and treatment preference of childhood obesity among the parents and their children.

This study is limited by the small sample size due to the uniqueness of the study group. Moreover, while the matched study design accounted for the impact of sex and age, other variables were uncontrolled. There were, however, no other group differences in perinatal, clinical or sociodemographic parameters. It can therefore be assumed that the differences that were found are attributable to different perceptions of “bariatric families”, a subject that needs further exploration. Those families are becoming more common in the pediatric nutrition clinics, and healthcare professionals should be aware of the differences between them and non-bariatric families and take those differences into account while planning treatment options. Since a parent’s perception has an important impact on the child’s success in losing weight, it is important to identify the children who are likely to fail to lose weight by standard dietary and lifestyle methods in order to avoid sense of failure and the metabolic effects of weight regain.

## Conclusion

This study is one of the first to define “bariatric families” and to demonstrate significant differences between the perceptions of those parents and children toward themselves, their health, and the preferred mode of obesity treatment compared to non-bariatric families. Larger studies are warranted in order to better comprehend the uniqueness and address the special needs of these families.

## Data Availability

The datasets used and/or analyzed during the current study are available from the corresponding author on reasonable request.

## Abbreviations

PedsQL: Pediatric Quality of Life Inventory
BMI: Body mass index
IQR: Interquartile range
SD: Standard deviation
